# The material-weight illusion is a Bayes-optimal percept under competing density priors

**DOI:** 10.7717/peerj.5760

**Published:** 2018-10-11

**Authors:** Megan A.K. Peters, Ling-Qi Zhang, Ladan Shams

**Affiliations:** 1 Department of Bioengineering, University of California, Riverside, Riverside, CA, United States of America; 2 Department of Psychology, University of California, Riverside, Riverside, CA, United States of America; 3 Interdepartmental Graduate Program in Neuroscience, University of California, Riverside, Riverside, CA, United States of America; 4 Department of Psychology, University of Pennsylvania, Philadelphia, PA, United States of America; 5 Department of Psychology, University of California, Los Angeles, Los Angeles, CA, United States of America; 6 Department of Bioengineering, University of California, Los Angeles, Los Angeles, CA, United States of America; 7 Neuroscience Interdepartmental Program, University of California, Los Angeles, Los Angeles, CA, United States of America; 8 Brain Research Institute, University of California, Los Angeles, Los Angeles, CA, United States of America

**Keywords:** Material-weight illusion, Visuohaptic perception, Size-weight illusion, Bayesian hierarchical causal inference, Heaviness perception

## Abstract

The material-weight illusion (MWI) is one example in a class of weight perception illusions that seem to defy principled explanation. In this illusion, when an observer lifts two objects of the same size and mass, but that appear to be made of different materials, the denser-looking (e.g., metal-look) object is perceived as lighter than the less-dense-looking (e.g., polystyrene-look) object. Like the size-weight illusion (SWI), this perceptual illusion occurs in the *opposite* direction of predictions from an optimal Bayesian inference process, which predicts that the denser-looking object should be perceived as *heavier*, not lighter. The presence of this class of illusions challenges the often-tacit assumption that Bayesian inference holds universal explanatory power to describe human perception across (nearly) all domains: If an entire class of perceptual illusions cannot be captured by the Bayesian framework, how could it be argued that human perception truly follows optimal inference? However, we recently showed that the SWI can be explained by an optimal hierarchical Bayesian causal inference process (Peters, Ma & Shams, 2016) in which the observer uses haptic information to arbitrate among competing hypotheses about objects’ possible density relationship. Here we extend the model to demonstrate that it can readily explain the MWI as well. That hierarchical Bayesian inference can explain both illusions strongly suggests that even puzzling percepts arise from optimal inference processes.

## Introduction

In general, much of human perception—including illusions—is well described by optimal computations. For example, visual percepts of motion (Weiss, Simoncelli & Adelson, 2002) are well described by optimal inference, despite sometimes leading to biased inferences about the structure of the environment. Likewise, the brain’s ability to combine information from multiple sensory modalities into an integrated percept also appears optimal, even though it too can lead to illusory percepts. When receiving multisensory information about a stimulus’ spatial location (Körding et al., 2007; Wozny, Beierholm & Shams, 2010), numerosity (Shams, Ma & Beierholm, 2005; Wozny, Beierholm & Shams, 2008), or body ownership (Samad, Chung & Shams, 2015) illusory percepts are also well explained by optimal inference.

Until recently, however, there remained a class of weight-related visuo-haptic illusions that appeared to defy optimality (Buckingham, 2014), calling into question whether human perception is in fact mathematically optimal. Two such illusions are the size-weight illusion (SWI), in which the smaller of two equal-mass objects is perceived as feeling heavier (Murray et al., 1999), and the material-weight illusion (MWI), in which an object appearing to be made of a denser material is perceived as feeling lighter than an object that looks like it is made of a less dense material (Harshfield & DeHardt, 1970). These weight-related multisensory illusions have puzzled psychologists for centuries because they appear “anti-Bayesian” (Ernst, 2009; Brayanov & Smith, 2010): rather than the ultimate percept demonstrating a weighted combination of expectations and incoming sensory information, the percept is in the “wrong direction”. Thus, a blanket conclusion that human perception is optimal is marred by these outliers, potentially suggesting an alternative computation underlying perceptual processing that may in some cases resemble Bayesian inference but in other cases does not.

To address this apparent discrepancy, we recently showed that one of these puzzling illusions—the SWI—can in fact result from an optimal inference strategy. Taking inspiration from previous literature on competitive priors in vision (Yuille & Bülthoff, 1996; Knill, 2003; Knill, 2007), we suggested that incoming haptic sensory information from two equal-mass but different-sized objects will arbitrate among competing density hypotheses, ultimately resulting in the illusory percept that the smaller item feels heavier than the larger one (Peters, Ma & Shams, 2016). This is because the incoming sensory information about the objects’ weight relationship (i.e., the two items actually have the same mass) is too incongruent with the *expected* weight relationship if they do in fact have the same density (i.e., that the smaller item should be lighter); instead, the alternative hypothesis that the smaller item is denser (Balzer, Peters & Soatto, 2014; Peters, Balzer & Shams, 2015) is determined to be *a posteriori* more probable, leading to its selection and the ultimate percept that the smaller item feels heavier. Using a series of behavioral experiments, we demonstrated this optimal computational framework well describes human perception (Peters, Ma & Shams, 2016).

However, it is important to note that other potential explanations for the SWI have been put forth (Anderson, 1970; Ross & Di Lollo, 1970; Cross & Rotkin, 1975; Ellis & Lederman, 1993; Brayanov & Smith, 2010; Wolf, Bergmann Tiest & Drewing, 2018). One possible way to arbitrate among these potential explanatory frameworks would be to demonstrate their generalizability to other weight illusions, such as the MWI. Here we use a series of simple simulations to show that the exact same competitive priors framework that explains the SWI (Peters, Ma & Shams, 2016) can also easily explain the MWI. This suggests that competing priors can provide a unifying framework for understanding even surprising multisensory percepts across a number of domains.

## Methods

### Description of behavioral effect

To induce the MWI, two same-volume objects appearing to be made of materials with different densities are presented to an observer simultaneously, and the observer is asked to lift them (simultaneously or sequentially) and judge their relative heaviness (Ellis & Lederman, 1999; Buckingham, Cant & Goodale, 2009; Baugh et al., 2012; Buckingham, 2014). In reality, both objects are constructed in such a way so that their physical mass is identical, despite their outward appearance; upon lifting the objects, the observer typically reports that the denser-*looking* object actually feels lighter than the other object. This MWI is generally of smaller magnitude than the related SWI (Buckingham, 2014), but otherwise demonstrates many similar properties and is robust to feedback training.

### Computational model

The computational model presented here is a direct application of the modeling framework previously developed to explain the SWI (Peters, Ma & Shams, 2016), but with parameters and settings modified to accommodate the circumstances of the MWI instead. Below we describe the logic of the model, and the modifications that were made to convert the model’s predictions from mapping the SWI to mapping the MWI.

#### Model intuition

**Figure 1:**
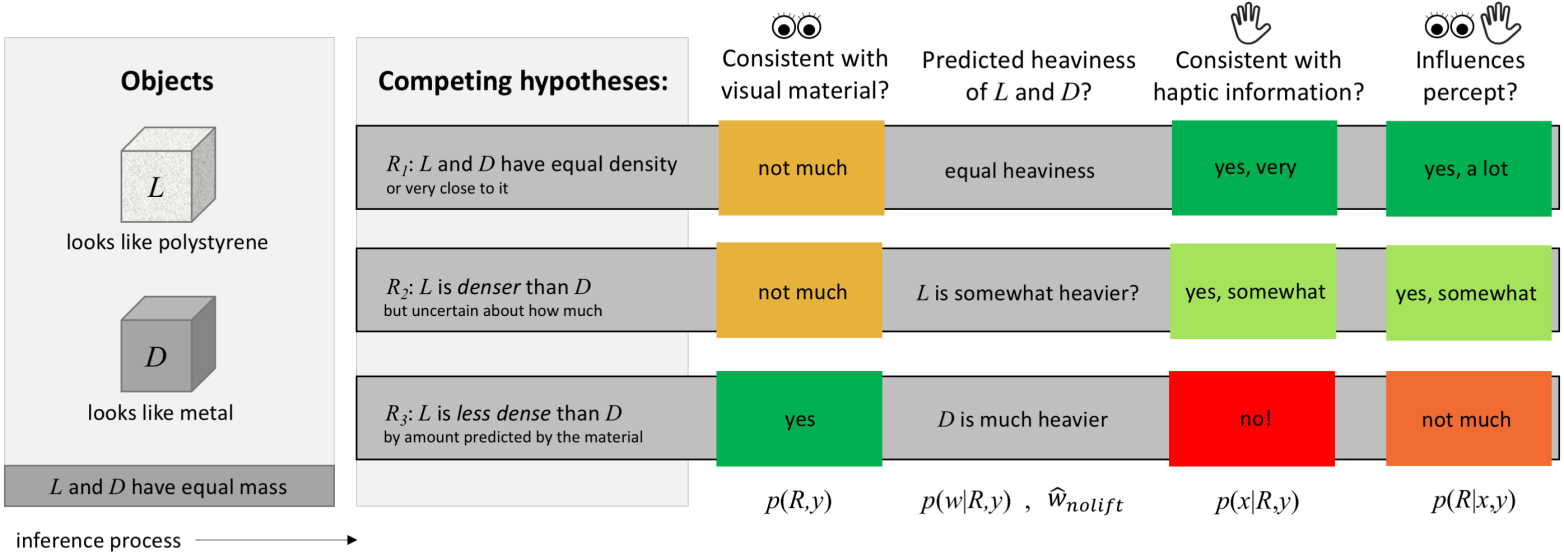
Flowchart intuition for the hierarchical Bayesian causal inference model. Proceeding from left to right, the inference process begins as the observer views two objects of the same size but different visual materials, *L* (appearing to be less dense) and *D* (appearing to be more dense). The observer is then tasked with evaluating the most likely heaviness relationship between them, given the possible competing density relationships (hidden or latent variables). The observer infers the *a posteriori* probability of each density relationship with respect to incoming sensory information via the haptic modality, and then weights the heaviness predictions from each competing density scenario according to these computed probabilities in order to arrive at the ultimate visuo-haptic percept of heaviness. Simulation results demonstrate that the competing density priors (hierarchical Bayesian inference) model can explain the MWI.

The foundation of the model is the assumption that the brain arbitrates among three possible density relationships between two objects using noisy haptic information, and that this arbitration process results in the observer illusorily perceiving the denser-looking object to be lighter than the less-dense-looking object. Although the MWI has previously been described in terms of single-lift events, fundamentally it is a comparison between a currently-lifted object and a comparison object, real or imagined—just as with the SWI (Peters, Ma & Shams, 2016). Thus, we formulate the model in terms of a comparison in the felt heaviness of two objects. The following paragraphs refer to Fig. 1 as a simplified, cartoon version of the competitive priors logic.

For clarity of explanation, we first designate the denser-looking object to be *D*, and the less dense-looking object to be *L*. The competing density relationships *R* we designate as *R*_1_, *R*_2_, and *R*_3_, where the subscript denotes the possible density relationship between the two objects: either they have equal density (*R*_1_), *D* is less dense than *L* (*R*_2_), or *D* is denser than *L* (*R*_3_).

Upon seeing two objects, each appearing to be made of a certain material, before lifting them an observer will form an expectation of the objects’ relative densities based on visual material estimate (Harshfield & DeHardt, 1970; Buckingham, Cant & Goodale, 2009; Buckingham, Ranger & Goodale, 2011), and the objects’ size (Peters, Balzer & Shams, 2015). For example, seeing two objects with the same size, one (*L*) appearing to be made of polystyrene and the other (*D*) appearing to be made of wood, the perceptual system ought to expect the wood-looking object to be quite a bit denser than the polystyrene-looking one. This expected relationship can be expressed as a ratio, e.g., 1:5, or the density of the polystyrene-looking object is five times less than that of the wood-looking object. The perceptual system will have some degree of uncertainty in this expected density relationship. Combined with the objects’ visually-estimated sizes (with some visual sensory noise), this density relationship estimate will lead to an expected weight relationship between the two objects; if the objects have the same volume, the density relationship 1:5 would lead to an expected weight relationship of 1:5 on average. Once the observer reaches out and lifts the two objects, either simultaneously or sequentially, a haptic estimate of the objects’ mass relationship (with some haptic sensory noise) will become available. If the objects physically have the same mass, as in the MWI (or SWI), the ratio of their masses is 1:1, meaning the noisy haptic estimate will be on average a ratio of 1:1. This sensory estimate of mass is combined with the expectations of weight given visual estimates of material and volume to lead to the ultimate percept of heaviness.

Crucially, in the competitive priors framework, the expected density relationship itself is uncertain not only in its magnitude, but in its quality, namely, the qualitative relationship between the density of *L* and *D*: *R*_1_, *R*_2_, and *R*_3_. The perceptual system assigns the highest probability (e.g., 0.8) to scenario *R*_3_ *a priori* because wood is often denser than polystyrene, but would also allow the possibility for the alternative scenarios (that for example, some clever experimenter or other unlikely circumstance has led them to have the *same* density or even that the wood-looking one may be hollowed out and thus be less dense). Each of these three qualitative density relationships also predicts an expected weight relationship between the two objects. If the objects have equal density, then they ought to weigh essentially the same (because they are the same size). If the wood-looking object has been hollowed out, then perhaps it is actually quite a bit less heavy than the polystyrene-looking object, leading to an expected weight relationship of e.g., 5:1—but with a much higher degree of uncertainty, to account for the fact that the observer has no idea what might have been done to lead to such a deviation from expectations. Because the *a priori* probability of each of these three scenarios is non-zero, they all influence the overall percept.

The key to explaining the MWI is the relative agreement between Eq. (1) the expectations of heaviness under the three competing density relationships, and Eq. (2) the incoming sensory information from the haptic modality. The percept is essentially a weighted average of the combined haptic information and competing expectations, where the ‘weight’ is determined by how much each expectation agrees with the haptic sensory information.

#### Model details

Because all the relationships can be expressed as ratios, we express these relationships in log space to ensure symmetry following previous convention (Sanborn, Mansinghka & Griffiths, 2013; Peters, Ma & Shams, 2016). There should be no difference between expressing the weight relationship when *L* feels heavier than *D* than when *D* feels heavier than *L*. The assumed density relationship of the objects under each *R*, $d=ln( \frac{{d}_{L}}{{d}_{D}} )$, together with the objects’ volume relationship, $v=ln( \frac{{v}_{L}}{{v}_{D}} )$, jointly determine the brain’s expectation of the weight relationship between objects prior to lifting them. Then, upon lifting the objects, the haptic information samples the objects’ true weight relationship, $w=ln( \frac{{w}_{L}}{{w}_{D}} )$. The incoming sensory information about *v* and *w* is assumed to be Gaussian, such that *p*(*x*|*w*) ∼ *N*(*w*, *σ*_*x*_) describes the noisy haptic estimate *x*of the objects’ weight relationship, and *p*(*y*|*v*) ∼ *N*(*v*, *σ*_*y*_) describes the noisy visual estimate *y* of the objects’ volume relationship. In both cases in the MWI, *v* = *w* = ln (1) = 0 because the objects do physically possess the same volume and same mass. (The astute reader will note that here we have elided the known bias in volume estimation previously used to model the SWI, i.e., that *v*^∗^ = *v*^.704^ (Peters, Ma & Shams, 2016), because in the MWI the two objects’ volumes are the same so it does not factor in to the ultimate weight percept.)

In modeling the SWI, we assumed that the appearance of the same surface material led to a strong prior probability that the two objects had the same density, i.e., that *R*_1_ was *a priori* highly probable (Peters, Ma & Shams, 2016). However, in the MWI the appearance of different materials leads to a strong *a priori* probability for *R*_3_, i.e., a strong belief that *D* should be denser than *L* before lifting the objects.

As in the SWI model, because the weight of an object is deterministically defined by the combination of its volume and density, we assume the probability of a given weight relationship between two objects given their density relationship and volume relationship, *p*(*w*|*v*, *d*), to be a delta function, *δw* − (*d* + *v*), which is defined as 0 at all impossible combinations of volume and density for a given weight relationship. Thus, this posterior mean of the log weight ratio under density hypothesis *R*_*i*_ will be

(1) \begin{eqnarray*}{\hat {w}}_{i}=\int \nolimits wp(w{|}{R}_{i},x,y)dw\end{eqnarray*}

When no haptic information is available (i.e., before the objects are lifted), the expected weight relationship between two objects can be computed as

(2) \begin{eqnarray*}{\hat {w}}_{nolift}=\int \nolimits wp(w{|}{R}_{i},y)p(R,y)dRdw\end{eqnarray*}

We use a joint prior over the density relationship *R* and volume estimation *y*, since usually in the daily environment they are highly correlated and the brain seems to be capable of utilizing this kind of natural statistic (Peters, Balzer & Shams, 2015). We indeed used some version of a joint Gaussian prior in modeling the SWI (Peters, Ma & Shams, 2016); however, in the MWI, since the density relationship is explicitly suggested by the visual material and not implicitly suggested by each object’s size, we assume these are independent of each other.

When haptic information is available, we first compute a posterior over the possible density relationship between objects via

(3) \begin{eqnarray*}p(R{|}x,y)= \frac{p(x{|}R,y)p(R,y)}{p(x)} \end{eqnarray*}

where the likelihood of the estimated haptic information under each density relationship, *p*(*x*|*R*, *y*), is given by

(4) \begin{eqnarray*}p(x{|}R,y)=\int \nolimits p(x{|}w)p(w{|}R,y)dw\end{eqnarray*}

As with the SWI, we obtain a single point estimate of the *perceived* weight relationship between the two objects given visual and haptic information, $\hat {w}$. This is accomplished via the mean of the posterior, i.e.,

(5) \begin{eqnarray*}{\hat {w}}_{lift}=\int \nolimits \int \nolimits wp(w{|}{R}_{i},x,y)p({R}_{i}{|}x,y)dRdw.\end{eqnarray*}

### Simulating behavioral data

We used simulations to demonstrate that the above-described hierarchical Bayesian inference model produces the MWI. To determine the inferred density relationship of objects used to induce the MWI and their true mass relationship, we set the relevant parameters directly according to the actual stimuli (and assumed densities, given visual material properties) used by Buckingham and colleagues (2009) in their Experiments 1 and 2, to show that the competitive density priors model can replicate the behavioral results demonstrated in their paper. We also present predictions of the model, which can be empirically tested in future studies.

Setting the empirical parameters of the objects’ apparent material (i.e., inferred density relationship) and true mass relationship leaves a number of free parameters, for which we assign reasonable values in this simple proof of concept; these values are reasonable because they (a) are similar to values used previously in a similar framework to explain the SWI (Peters, Ma & Shams, 2016), (b) are consistent with the intuition that prior expectations ought to be less reliable than incoming sensory information, and (c) utilize the natural statistics of the environment regarding objects’ weights and density relationships (Peters, Balzer & Shams, 2015). Global parameter values used in producing all simulation results presented here are shown in Table 1; individual parameter values used in reproducing Buckingham and colleagues’ (2009) Experiments 1 and 2 are presented in each section below. All Monte Carlo simulations use 100,000 trials in each condition, and were conducted in the high-level probabilistic language Church.

**Table 1:** Parameter values used in all simulations. All code is available at https://github.com/zlqzcc/WeightIllusionChurch/blob/master/MWIsimulation.lisp.

**Parameter name**	**Symbol**	**Value**
Prior probability of equal density	*p* (*R*_1_)	0.1
Prior probability that *D* is less dense than *L*	*p* (*R*_2_)	0.1
Prior probability that *D* is denser than *L*	*p* (*R*_3_)	0.8
Expected density relationship assuming equal density (*R*_1_)	*d*_1_	0
Expected density relationship assuming *D* is less dense than *L* (*R*_2_)	*d*_2_	0.6
Expected density relationship assuming *D* is denser than *L* (*R*_3_)	*d*_3_	Table 2
Uncertainty in density relationship assuming equal density (*R*_1_)	*σ*_*R*_1__	0.1
Uncertainty in density relationship assuming *D* is less dense than *L* (*R*_2_)	*σ*_*R*_2__	0.6
Uncertainty in density relationship assuming *D* is denser than *L* (*R*_3_)	*σ*_*R*_3__	0.1
True density relationship between the objects	$d=ln \left( \frac{{d}_{L}}{{d}_{D}} \right) $	0
True volume relationship between the objects	$v=ln \left( \frac{{v}_{L}}{{v}_{D}} \right) $	0
True weight relationship between the objects	$w=ln \left( \frac{{w}_{L}}{{w}_{D}} \right) $	0 (Sims 1 & 3); Table 3 (Sim 2)
Uncertainty in visual estimate	*σ*_*y*_	0.31
Uncertainty in haptic estimate	*σ*_*x*_	0.4

#### Simulation 1: Buckingham, Cant & Goodale’s (2009) Experiment 1

In their Experiment 1, to induce the MWI Buckingham and colleagues (2009) presented observers with 700 g objects that appeared to be made of one of three materials: solid aluminum (density: ∼2,700 kg/m^3^), polystyrene (density: ∼100 kg/m^3^), or wood (density: ∼700 kg/m^3^). This leads to three possible density ratios under *R*_3_, dictated by the visual cues to material (Table 2).

#### Simulation 2: Buckingham, Cant & Goodale’s (2009) Experiment 2

In their Experiment 2, Buckingham, Cant & Goodale (2009) modified their original stimuli such that the wood block still weighed 700 g, but the polystyrene block now weighed 680 g and the aluminum block now weighed 720 g. They then repeated their MWI experiment. To mimic this approach, we modified the mean of the haptic likelihood function, i.e., the mean of *p*(*x*|*w*), to reflect the true ratio of the objects’ weights in this experiment (Table 3). All other parameters remain the same as used in Simulation 1.

#### Simulation 3: the effect of changing the expected density ratio under R_2_

The competitive priors framework makes an additional prediction regarding what should happen as a function of training. We previously showed that the competitive density priors model can account for the “inversion” of the SWI that occurs as a result of training with “large-heavy, small-light” objects (Flanagan, Bittner & Johansson, 2008; Ernst, 2009); this is because as the prior for expected density ratio given “smaller is denser” is modified such that it becomes *too* incongruent with incoming haptic information, other possible density relationships become *a posteriori* more probable and ultimately drive the percept (Peters, Ma & Shams, 2016). The same phenomenon may occur with the MWI: if, through training or other modification, observers learn that the less-dense-looking object should actually be *much* denser than the denser-looking object (i.e., under *R*_2_), the model should predict that the illusion may ultimately attenuate and be reversed, just as with the SWI. To show this prediction in simulation, we used the same parameter values used in Simulation 1, but incremented the expected density relationship assuming *D* is less dense than *L* (*R*_2_) from (used in Simulation 1) to 2.

## Results

### Simulation 1: Buckingham, Cant & Goodale’s (2009) Experiment 1

**Figure 2:**
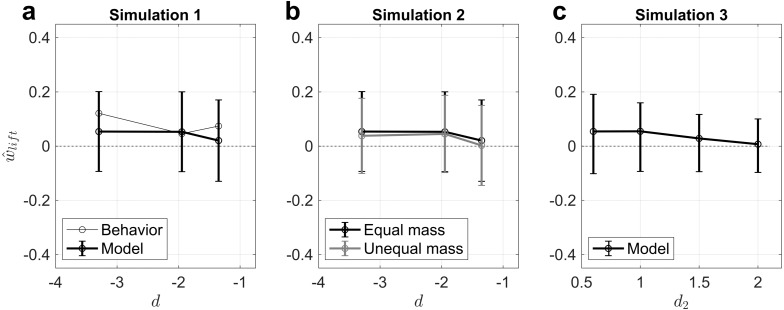
Simulation results demonstrate that the competing density priors (hierarchical Bayesian inference) modeling framework can explain the MWI. (A) The magnitude of the MWI for three object pairs is in line with that reported by Buckingham and colleagues (2009). (B) The MWI for all three object pairs is attenuated when the true weight of the objects is experimentally manipulated, as shown previously (Buckingham, Cant & Goodale, 2009). (C) If the expected density ratio given that the denser-looking object is actually *less* dense (*R*_2_) is manipulated, e.g., through training, the model predicts an attenuation of the MWI. In all plots, error bars refer to the standard deviation (not standard error) across 100,000 Monte Carlo simulations, but should not be taken to result from the number of samples used; the variability in the estimate is instead a consequence of noise terms in the model and the inference process.

To compare the behavioral results reported by Buckingham and colleagues (2009) in their Experiment 1 to our model, we first translated their behavioral results into ratios using the raw data (G Buckingham, pers. comm., 2018). Our model’s predicted MWI magnitude is in qualitative agreement with the MWI magnitudes reported by Buckingham, Cant & Goodale (2009), albeit with some differences in predicted order of MWI magnitude as a function of material pair that may be unaccounted for by the simplicity of our model (Fig. 2A, Table 4). Nevertheless, that our competing density priors model can produce MWI magnitudes qualitatively in line with MWI magnitudes observed in the literature supports the theory that the MWI, like the SWI, may occur as a result of optimal inference over competing density priors.

**Table 2:** Assumed density ratios under *R*_3_, as dictated by visual cues to material, following stimuli used by Buckingham and colleagues (2009) in their Experiment 1.

**Material of*L***	**Material of*D***	$d=\ln \left( \frac{{d}_{L}}{{d}_{D}} \right) $
polystyrene	aluminum	−3.296
polystyrene	wood	−1.946
wood	aluminum	−1.350

**Table 3:** Means of haptic likelihood functions *p*(*x*|*w*) given that the objects with different visual material properties actually do *not* have the same mass, as in Buckingham and colleagues’ (2009) Experiment 2.

**Material & Mass of*L***	**Material & Mass of*D***	$w=\ln \left( \frac{{w}_{L}}{{w}_{D}} \right) $
polystyrene (680 g)	aluminum (720 g)	−0.057
polystyrene (680 g)	wood (700 g)	−0.029
wood (700 g)	aluminum (720 g)	−0.028

**Table 4:** Results from the competitive density priors hierarchical inference model. The results are in close agreement with the magnitude of the MWI reported by Buckingham and colleagues (2009).

**Material of*L***	**Material of*D***	${\hat {w}}_{lift,behavior}$	${\hat {w}}_{lift,model}$
polystyrene	aluminum	0.121	0.053
polystyrene	wood	0.046	0.056
wood	aluminum	0.074	0.021

### Simulation 2: Buckingham, Cant & Goodale’s (2009) Experiment 2

To evaluate whether our model could also explain Buckingham, Cant & Goodale’s (2009). Experiment 2, we then changed the ratio of true masses in the model to match their stimulus manipulations (see ‘Methods’). All other parameters were identical to those used in Simulation 1. Buckingham and colleagues (2009) reported that the manipulation of objects’ actual mass led all three objects to be judged of similar heaviness, which would translate into ${\hat {w}}_{\mathrm{lift},\mathrm{behavior}}=0$. Our model’s predictions agree with this finding (Fig. 2B), showing ${\hat {w}}_{lift}$ is attenuated—and indeed becomes nearly 0, i.e., no illusion, for the wood:aluminum pair—for all conditions we tested. This provides additional evidence in support of the competing density prior model for explaining the MWI.

### Simulation 3: The effect of changing the expected density ratio under *R*_2_

To explore the model’s predictions for future experimental studies, we also evaluated what would happen if participants learned, e.g., through training (Flanagan, Bittner & Johansson, 2008; Ernst, 2009), that denser-looking objects are in fact *less dense* by an extreme amount. We estimated four different levels of such ‘training’ for the MWI magnitude for the ‘most unequal density’ pair (polystyrene:aluminum), because this pair ought to maximize the salience of any effect, as the expected density ratio under *R*_2_ became increasingly extreme. Because the effect of this ‘training’ in the competitive prior framework is to make *R*_2_ in increasing disagreement with incoming haptic sensory information, the model predicts an increasingly attenuated MWI magnitude (Fig. 2C). Importantly, no reversal is predicted because one of the competing density priors—the ‘equal density’ prior, *R*_1_—predicts an expected weight relationship (before lifting) that is centered at 0 because the objects are the same size. As a result, with training, this is the competing prior expectation that ends up “winning” even more strongly, and so the predicted percept is that because the expectation and the incoming sensory information both lead to the conclusion that the objects have the same mass, the objects will feel equal in weight (which they are!). Interestingly, this observation may explain why the MWI is predicted a priori to be weaker in magnitude than the SWI, which is borne out in the empirical data throughout the literature (Buckingham, 2014); we return to this point in the Discussion.

### Robustness of model to parameter value variation

**Figure 3:**
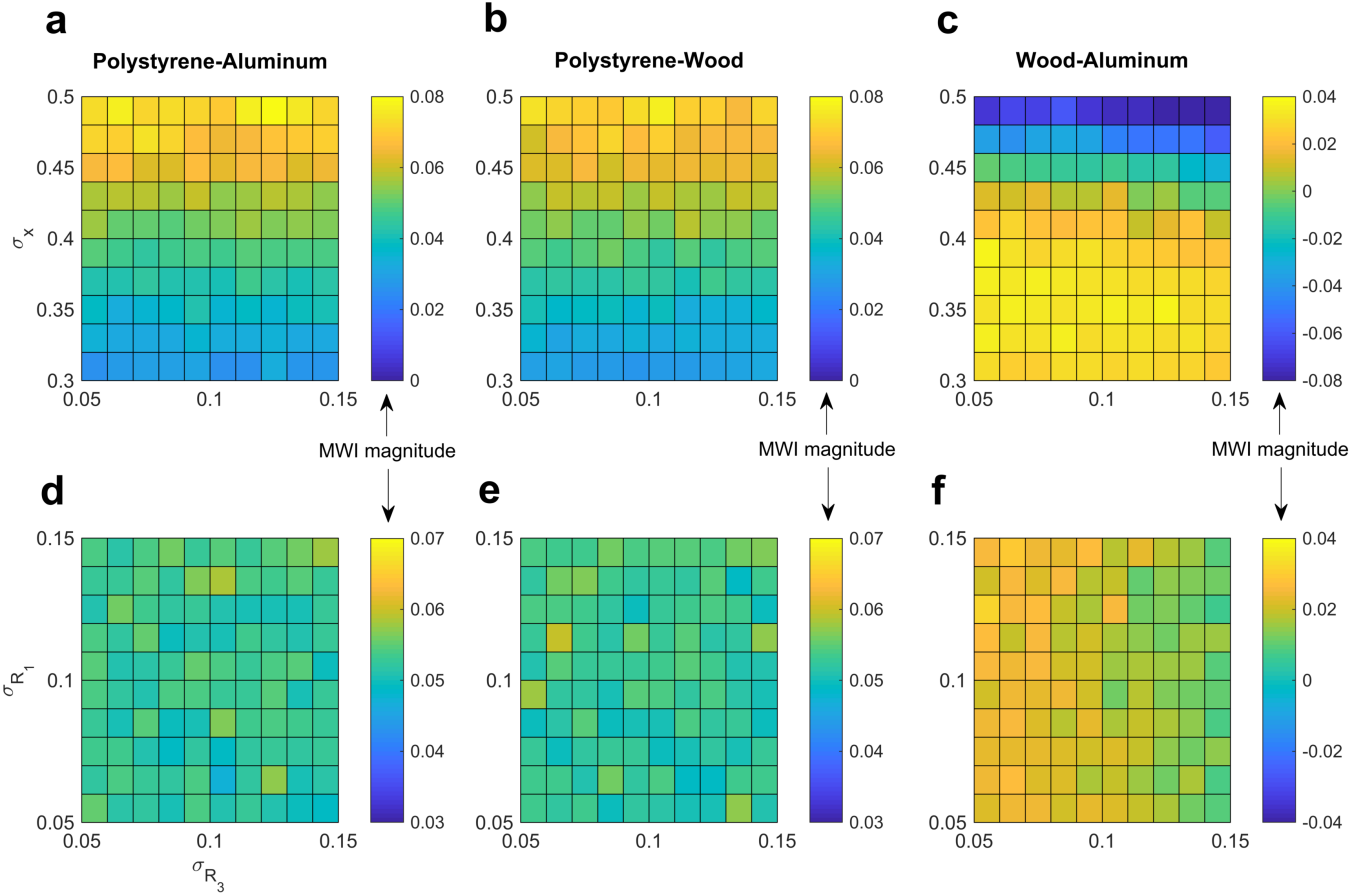
Robustness of model predictions to variation in parameter value selection. By varying the reliability of the haptic information and the expectations for weight under different density scenarios, we demonstrate that the presence and magnitude of the illusion is relatively robust to quite large variations in parameter value selection. In particular, we show that the reliability of weight expectations has relatively smaller effect than the reliability of haptic information. (A, B, C) show the effect of varying haptic reliability and that of weight expectations under *R*_3_, when the denser-looking object *D* is assumed denser than the less-dense-looking object *L*. (D, E, F) show the effect of varying reliability of weight expectations under equal density assumptions, *R*_1_, as well as *R*_3_.

Importantly, we note that a large range of parameter values produces qualitatively similar results. To illustrate this property of the model, we calculated predicted MWI magnitudes under a range of reliabilities for haptic and *a priori* expectations about the objects’ weight relationships. Under a large range of conditions the model predicts the presence of the MWI, in relatively stable magnitude (Fig. 3).

Finally, to demonstrate the robustness of the model to quantitative perturbations in parameter values, we also release the code used to create these predictions, written in the high-level probabilistic language Church; we invite any interested researchers to explore the model’s predictions under different sets of assumptions.

## Discussion

Here we showed that the MWI can be explained by the exact same computational framework that neatly accounts for the SWI. First, using parameters set to experimental stimuli used in the literature, i.e., Experiment 1 as reported by Buckingham and colleagues (2009), we demonstrated that the model’s predicted MWI magnitude is in line with the MWI magnitude reported by human subjects. To challenge the model, we then modified the mean of the haptic likelihood function in the model to account for the physical modifications to stimuli mass the same authors used in their Experiment 2. The model predicts that the perceived weight of all three objects should be closer to an equal-weight estimate, which is again qualitatively consistent with behavioral findings reported in the original study (Buckingham, Cant & Goodale, 2009). These results support the interpretation that hierarchical Bayesian inference may represent a unifying framework across a broad range of perceptual domains (Shams, Ma & Beierholm, 2005; Körding et al., 2007; Wozny, Beierholm & Shams, 2008; Wozny, Beierholm & Shams, 2010; Samad, Chung & Shams, 2015; Peters, Ma & Shams, 2016).

We also showed that our model predicts that training or other manipulations of the learned density expectations given material cues might lead to attenuation (and possibly even reversal, under some instances) of the MWI. This is akin to the training manipulation done by Flanagan, Bittner & Johansson (2008) for the SWI, wherein participants learned that small objects were very dense and large objects were not dense. We believe the results presented here are in line with other competing prior explanations of weight illusions, which have shown that well-learned perceptual priors linking an object’s visual properties (typically size) can be updated through experience lifting objects that violate those expectations (Baugh et al., 2016). Future studies should test whether training with objects that violate expected density given material cues would lead to attenuation and possibly reversal of the MWI.

We do note that there are differences between our model’s predictions and reported behavior (Buckingham, Cant & Goodale, 2009) both in the magnitude of the illusion for some conditions and the ‘order’ of MWI magnitude as a function of visual cues to material. This divergence between model and behavior may occur because the model predicts that as the density ratio predicted by the material cue (*R*_3_) becomes less extreme (i.e., the difference predicted by wood:aluminum is less than the difference predicted by polystyrene:wood), the illusion magnitude should attenuate, but this was not observed in the behavior. Because our model does not account for also possible differences in motor force application often observed in weight illusions (Buckingham, 2014), it may not be able to capture the effects of motor force application or other factors on the illusion magnitude. Another possible source of the discrepancy is that the true densities of various materials may not reflect an observer’s judgment of likely density, especially since it has been shown that human observers are sensitive to environmental density fluctuations also dependent on an object’s size (Peters, Balzer & Shams, 2015). More work is needed to better elucidate how these and other factors may impact MWI magnitude and to fit the model’s parameters to behavioral data.

The primary goal of this study was to demonstrate that the same modeling framework that previously was shown to account for the SWI (Peters, Ma & Shams, 2016) can also account for the MWI. To facilitate explorations of the model’s predictions and quantitative data fitting, we have released the code used in all the simulations demonstrated here—written in the high-level language Church and available at https://github.com/zlqzcc/WeightIllusionChurch/blob/master/MWIsimulation.lisp. We invite any interested researchers to make use of it with their own data, to fit the model and/or test its predictions.

Importantly, despite the similarities in the modeling framework and in the surprising, counterintuitive nature of both the SWI and MWI, the two illusions do demonstrate important differences. The primary difference that has been noted extensively is that the magnitude of the MWI is significantly smaller than that of the SWI (Buckingham, 2014). We believe a comparison between the modeling results shown here and for the SWI previously (Peters, Ma & Shams, 2016) provides a potential explanation for this difference. With the SWI, the competition among possible density relationships is arbitrated quite decisively by the incoming haptic information: the haptic-only estimate that the two objects have the same mass is highly consistent with the smaller item being denser than the larger one (because it actually is), and highly *inconsistent* with the possibility that they have the same density or that the larger item is denser. This leads to the “smaller is denser” relationship essentially “winning” the competition, i.e., being extremely probable *a posteriori*, and thus influencing the illusory visuohaptic percept the most. In contrast, in the MWI paradigm, the incoming haptic information is actually most consistent with the possibility that the two items have the same density; this haptic information is highly *inconsistent* with predictions from a density relationship dictated by the visual material, but remains somewhat compatible with the possibility that the denser-looking object is actually less dense because this possibility is extremely uncertain. Thus, the “equal density” (and thus equal heaviness) relationship is the one that “wins”, but a small amount of influence is still exerted by predictions from the “denser-looking is less dense” relationship. Thus, the SWI being larger in magnitude than the MWI is predicted by the competitive priors modeling framework.

Ours is certainly not the first model claiming to explain the origins of the puzzling MWI or other related weight illusions. Models positing a role for top-down processing in heaviness perception (Ross, 1969; Ellis & Lederman, 1998; Ellis & Lederman, 1999) or ‘anti-Bayesian’ biases (Brayanov & Smith, 2010) have been explored. Likewise, a number of early models claimed to describe especially the SWI (which is more robust and larger in magnitude than the MWI) in a cue combination framework by arbitrarily placing a negative weight on the expectancy cue (Anderson, 1970) or other similar assumptions (Cross & Rotkin, 1975). Many of these models were unsatisfactory primarily because they described the magnitude of weight illusions but did not *explain* why they occurred. In contrast, the competitive density priors model provides an explanatory framework through generative modeling—and here we have shown that this framework explains both the MWI and SWI as examples of an entire class of weight illusions.

A recent report (Wolf, Bergmann Tiest & Drewing, 2018) proposed an alternative generative model to explain the SWI, and one might wonder whether that model could better explain the MWI as well. Like other averaging models proposed previously (Anderson, 1970), their model proposed a reliability-weighted cue combination framework to combine mass and density estimates in producing a percept of heaviness in the SWI. However, their model could not account for the MWI and is also somewhat circular, in that the estimate of density by definition relies on estimates of mass and volume. In contrast, the competitive density priors model described here can explain both the SWI (Peters, Ma & Shams, 2016) and the MWI, as shown here. (We note also that the competitive prior model can also account for often-reported increase in the magnitude of the illusion due to an increase in the objects’ actual weight through a simple increase in the noisiness of the haptic estimate (*σ*_*x*_), as predicted by Weber’s law (Fechner, 1966)).

The competitive density priors framework leads to a number of interesting predictions for numerous weight illusions, including the possible influence of haptic precision (*σ*_*x*_), expected density ratios given visual cues to size or material, and alterations in these expectations due to training or other manipulations. For example, it could be argued that overlifting or underlifting due to a mismatch between expected weight of an object and its actual weight could add noise to the haptic estimate, potentially affecting results; however, perceptual weight illusions have traditionally been puzzling because they appear to defy such motor-based explanations, given that they persist long after motor forces have scaled appropriately to the true mass of a lifted object (Buckingham, Cant & Goodale, 2009;  Buckingham & Goodale, 2010;  Flanagan & Beltzner, 2000; Flanagan et al., 2001;  Grandy & Westwood, 2006) or when lifting style, rotational inertia, and grip size are controlled for (Ellis & Lederman, 1998). Another intriguing possibility is the supposition that participants’ conscious awareness of the mismatch between their expectations of an object’s weight and its actual mass might lead to a top-down discounting of haptic information. In this case, participants could potentially discount haptic information almost entirely, which might lead to conditions similar to providing expectations about objects’ heaviness before they are lifted. But participants typically rate expected weight based on the visual size and apparent material of an object (Buckingham, Ranger & Goodale, 2011), meaning that if they completely discounted their haptic information, they should experience an ‘inverted’ MWI, i.e., that the denser-looking object should feel heavier, not lighter. This discussion, and the results presented here, represents only a few possibilities as a proof of concept; we encourage other researchers in the field to make use of the code we have provided in critically evaluating the extent to which the competitive density prior model carries true explanatory power across a wide variety of weight illusions—beyond those presented here and previously (Peters, Ma & Shams, 2016).

We also think it is important to reiterate that the hierarchical modeling framework used here to describe heaviness illusions is not limited to this task. In the present study, we demonstrate the power of this type of model to reveal important patterns in how latent (i.e., unobservable) variables—including but not limited to objects’ density—may be represented in the brain. The present results we describe suggest that density relationships may be encoded in a categorical fashion, i.e., that one item is likely to be denser than another by some stereotypical amount with some noise. It is possible that other latent variables are also encoded categorically, perhaps for conservation of limited neural or computational resources, or that the way a latent variable is encoded may change depending on task, context, or other factors. Future studies should directly compare predictions from such categorical latent variable representations to those from continuously-represented variables (e.g., Peters, Balzer & Shams, 2015) to discover when and how the brain may conserve resources or processing power by simplifying representations into the categorical.

In sum, here we have demonstrated via simple simulations that competing density priors and hierarchical Bayesian causal inference can explain the MWI. Our results demonstrate that the MWI, like the SWI, no longer represents a challenging counterexample to the theory that human perceptual experience results from Bayesian inference.

## Additional Information and Declarations

### Data Availability

The following information was supplied regarding data availability: GitHub: https://github.com/zlqzcc/WeightIllusionChurch/blob/master/MWIsimulation.lisp.
